# Incidence of Chronic Obstructive Pulmonary Disease, and the Relationship between Age and Smoking in a Japanese Population

**DOI:** 10.2188/jea.17.54

**Published:** 2007-04-10

**Authors:** Shigeko Kojima, Hiroki Sakakibara, Shinichi Motani, Kunihiko Hirose, Fumio Mizuno, Masahiro Ochiai, Shuji Hashimoto

**Affiliations:** 1 Division of Hygiene, Department of Medicine, Fujita Health University.; 2 Division of Respiratory Medicine and Clinical Allergy, Department of Internal Medicine, Fujita Health University.; 3 Toyota Regional Medical Center.

**Keywords:** Pulmonary Disease, Chronic Obstructive, Incidence, Smoking, Epidemiology, Age Factors

## Abstract

**BACKGROUND:**

Accurately evaluating a risk of chronic obstructive pulmonary disease (COPD) requires a large-scale longitudinal study using a standard criterion for diagnosing COPD. There have been only a few such follow-up studies in Europe and no reports in Asia. We estimated the incidence rate and incidence rate ratio (IRR) of age and smoking for COPD in a Japanese population using the diagnosis criterion of the Global Initiative for Chronic Obstructive Lung Disease guidelines.

**METHODS:**

Subjects were 17,106 participants aged 25-74 years during health check-ups including spirometry from April 1997 through March 2005 in Japan. Total follow-up of participants were 47,652 person-years in males and 25,224 person-years in females. The IRR of age and smoking was estimated using Cox proportional hazard models with both variables.

**RESULTS:**

We identified 466 incidence cases of COPD. The incidence rate per 100 person-years was 0.81 (95% confidence interval [CI], 0.73-0.89) in males and 0.31 (0.24-0.38) in females, and significantly increased with age in both sexes. The incidence rate for current smokers was significantly higher than that for male non-smokers but not significantly for females. Among males, the IRR for current smokers with Brinkman Index < 400, 400-799, and 800+ was 1.2 (0.8-1.9), 2.7 (1.9-3.8), and 4.6 (3.3-6.5), respectively.

**CONCLUSION:**

These results indicated that the COPD risk gradually increased with aging, and that there was a dose-response relationship between smoking and COPD risk.

Many cross-sectional studies have reported the prevalence of chronic obstructive pulmonary disease (COPD) and its related factors such as age and smoking in Asia,^1^^-^^3^ Europe,^4^^-^^6^ and South America.^7^ However, accurately evaluating an incidence of COPD requires a large-scale longitudinal study using a standard criterion for diagnosing COPD. The incidence rate and incidence rate ratio (IRR) of age and smoking were examined from a limited number of longitudinal studies in Europe,^8^^-^^12^ and its epidemiologic evidence in Asia was still not available. Recently, the Global Initiative for Chronic Obstructive Lung Disease (GOLD) guidelines recommended a forced expiratory volume at one second per forced vital capacity (FEV_1_/FVC) < 70% for a pragmatic diagnosis criterion of COPD.^13^ Only a few longitudinal studies have been based on the standard criterion.^10^^-^^12^

The objectives of the present follow-up study were to estimate the incidence rate of COPD in a Japanese population using the standard criterion of the GOLD guidelines, and to evaluate the effects of smoking and age on COPD incidence.

## METHODS

### Subjects

Subjects in this study were participants aged 25-74 years subjected to health check-ups including spirometry from April 1997 through March 2005 at the Toyota Regional Medical Center in Japan. Out of 30,246 participants, 1,025 with asthma or tuberculosis were excluded because of the difficulty in diagnosing COPD by spirometry, 231 with COPD at baseline of each first check-up were excluded from the subject of follow up, 11,880 with only one health check-up and no information of follow-up were excluded, and 4 were excluded because of insufficient data. Thus, 17,106 participants were left for follow-up.

### COPD Diagnosis

The baseline information on subjects was obtained from the first health check-up. Follow-up information was obtained from their health check-ups after baseline in the study period between April 1997 and March 2005. Spirometry (DISCOM 21 FX II; CHEST MI., Ins., Tokyo, Japan) was performed by trained technicians in the health check-up, and the test procedure followed the American Thoracic Society recommendation.^14^ The calibration of the spirometric instrument was performed at least every testing day. FVC and FEV_1_ were measured, and FEV_1_/FVC was calculated. According to the standard criteria of the GOLD guidelines, subjects were diagnosed as COPD for FEV_1_/FVC <70% using their follow-up information.

### Smoking Status

A self-administered questionnaire was used to determine smoking status including the number of cigarettes per day and the years of smoking. Subjects were classified by smoking status at baseline into three groups: non-smokers, former smokers, and current smokers. Current smokers were classified by the Brinkman Index (BI)^15^ into three groups: BI <400, BI 400-799, and BI 800+. The BI was determined as the number of cigarettes per day multiplied by the years since smoking had started.

### Data Analysis

Data from the subjects mentioned above were available for sex, age, smoking status, BI, FVC and FEV_1_, but did not include any personal identifiers such as name or address. We counted person-year of follow-up for each subject from the date of the baseline of the first health check-up to incidence of COPD or the last health check-up in the study period. The date of incidence of COPD was determined as a median date between the health check-ups with the first diagnosis of COPD and with the last diagnosis of not having COPD. Those who were not diagnosed as COPD during the follow-up period were treated as censored cases.

The incidence rate of COPD by sex, age, smoking status, and follow-up period was calculated as the number of COPD incidence cases divided by the person-years of follow-up. The age was classified into 10 groups of 5-year intervals from 25-29 to 70-74 years old. The follow-up period was classified into less than 2 years and 2 years or more after baseline. The incidence rate ratios (IRRs) of age groups to 40-44 years old and those of smoking status to non-smokers by sex were estimated using Cox proportional hazard models with both variables. These analyses were performed using an SPSS^®^ 12.0J software package (SPSS Japan Inc.).

### Ethical Review

This study was approved on March 2005 by the Ethical Review Board for Epidemiological and Clinical Studies of the Fujita Health University School of Medicine.

## RESULTS

### Baseline Characteristics of Subjects

Table 1 shows the number of subjects by age group. The subjects were 11,160 males and 5,946 females, and their mean age was 47.7 years in males and 48.0 years in females. Table 2 shows the number of subjects by smoking status. The proportion of current smokers was 43.3% in males and 5.5% in females.

**Table 1.** Number of subjects and incidence cases for chronic obstructive pulmonary disease (COPD) and incidence rate by sex and age.

Age (years)	Males			Females		
	
	n	Number of incidence cases for COPD	Incidence rate (per 100 person-years)	n	Number of incidence cases for COPD	Incidence rate (per 100 person-years)

Total	11,160	387	0.81	5,946	79	0.31

25-29	94	2	0.62	36	0	0.00
30-34	625	7	0.31	181	1	0.16
35-39	1,609	24	0.35	712	4	0.13
40-44	1,973	45	0.47	1,161	10	0.18
45-49	2,153	65	0.61	1,279	12	0.19
50-54	1,879	90	1.05	1,157	21	0.42
55-59	1,729	74	1.25	1,002	12	0.35
60-64	745	39	1.67	264	9	1.02
65-69	252	24	2.75	109	7	1.69
70-74	101	17	4.95	45	3	2.05

**Table 2.** Number of subjects and incidence cases for chronic obstructive pulmonary disease (COPD) and incidence rate by sex and smoking status.

Brinkman Index^†^	Males			Females		
	
	n	Number of incidence cases for COPD	Incidence rate (per 100 person-years)	n	Number of incidence cases for COPD	Incidence rate (per 100 person-years)

Total	11,160	387	0.81	5,946	79	0.31

Non-smokers	2,784	52	0.44	5,416	74	0.32
Former smokers	3,541	111	0.70	201	0	0.00
Current smokers	4,835	224	1.14	329	5	0.44
<400	1,712	29	0.39	284	3	0.28
400-799	2,126	100	1.11	38	2	1.30
800+	997	95	2.64	7	0	0.00

**†** : number of cigarettes per day × total years smoking.

Total follow-up of participants were 47,652 person-years in males and 25,224 person-years in females, and the mean follow-ups were 4.3 (range, 0.3-8.0) years in males and 4.2 (0.3-7.9) years in females. The mean numbers of those receiving a health check-up in follow-ups were 3.6 (range, 1.0-9.0) times in males and 3.3 (1.0-8.0) times in females. Most subjects received a health check-up about once a year.

### Incidence Rate of COPD

We identified 466 incidence cases of COPD. The incidence rate of COPD per 100 person-years was 0.81 (95% confidence interval [CI], 0.73-0.89) in males and 0.31 (0.24-0.38) in females. Table 3 shows the incidence rate by follow-up period. The incidence rate for the follow-up period of less than 2 years after baseline and 2 years or more was 0.71 and 0.88 in males, and 0.31 and 0.32 in females, respectively.

**Table 3.** Person-years, the number of incidence cases for chronic obstructive pulmonary disease (COPD) and incidence rate by sex and follow-up period.

Follow-up period	Males			Females		
	
	Person-years	Number of incidence cases for COPD	Incidence rate (per 100 person-years)	Person-years	Number of incidence cases for COPD	Incidence rate (per 100 person-years)
Total	47,652	387	0.81	25,224	79	0.31

Less than 2 years after baseline	18,004	127	0.71	10,800	33	0.31

2 years or more after baseline	29,648	260	0.88	14,423	46	0.32

Table 1 shows the incidence rate by age group, which ranged between 0.31 and 4.95 in males and between 0.00 and 2.05 in females. Table 2 shows the incidence rate by smoking status. The incidence rate for non-smokers was 0.44 in males and 0.32 in females. The incidence rate for current smokers was higher than one for non-smokers. The incidence rate increased with BI in male current smokers.

**Figure 1.**
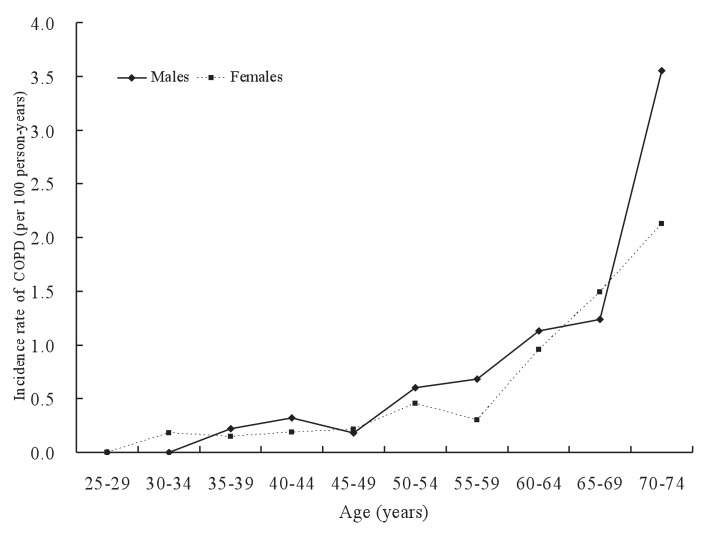
Incidence rates of chronic obstructive pulmonary disease (COPD) by age and sex among non-smokers.

The incidence rate by age among non-smokers is illustrated in Figure 1. The incidence rate per 100 person-years ranged between 0.00-0.32 for the age groups of 25-49 years. For the age groups of 50 years or more, it gradually increased with age from 0.61 to 3.55 in males and from 0.45 to 2.11 in females.

### Association between COPD Incidence, Age and Smoking

**Figure 2.**
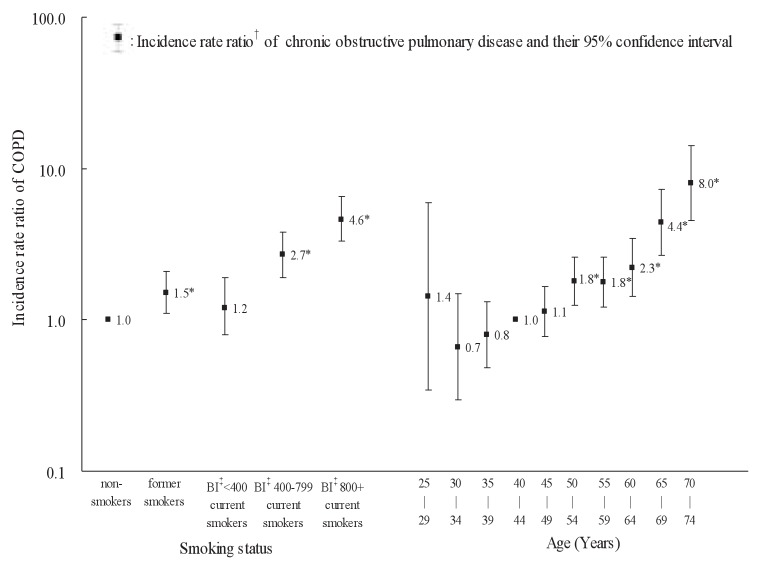
Incidence rate ratios of chronic obstructive pulmonary disease (COPD) by age and smoking among males. * : p <0 .01. † : Incidence rate ratios of age groups to 40-44 years old and those of smoking status to non-smokers were estimated using Cox proportional hazard models with an adjustment of the other variable. ‡ : Brinkman Index: number of cigarettes per day × total years smoking.

**Figure 3.**
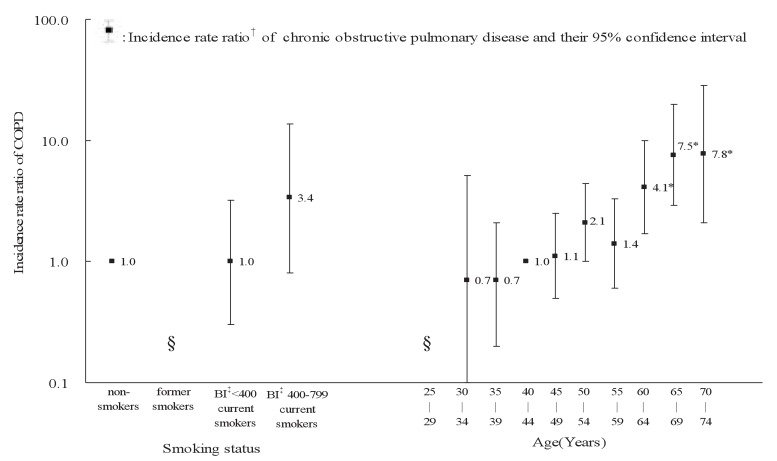
Incidence rate ratios of chronic obstructive pulmonary disease (COPD) by age and smoking among females. * : p < 0.01. † : Incidence rate ratios of age groups to 40-44 years old and those of smoking status to non-smokers were estimated using Cox proportional hazard models with an adjustment of the other variable. ‡ : Brinkman Index: number of cigarettes per day × total years smoking. § : not estimated.

The IRRs of age groups to 40-44 years old and those of smoking status to non-smokers adjusted for the other variable are illustrated in Figure 2 for males and in Figure 3 for females. The IRR significantly increased with age in males (p < 0.01) and in females (p < 0.01). Among males, the IRR for former and current smokers was significantly higher than one for non-smokers. The IRR (95% CI) for current smokers with BI <400, 400-799 and 800+ were 1.2, (0.8-1.9), 2.7 (1.9-3.8), and 4.6 (3.3-6.5), respectively. The trend in IRR over the BI categories was statistically significant (p < 0.01). Among females, the IRR for former smokers and current smokers with 800 or more of BI was not estimated due to no cases of COPD. The IRR for current smokers was not significantly higher than one for non-smokers.

## DISCUSSION

The present follow-up study identified 466 incidence cases of COPD using the standard criterion of the GOLD guidelines, and the incidence rate per 100 person-years was 0.81 in males and 0.31 in females. Previously, there have been only three follow-up studies with the GOLD-defined incidence of COPD in Europe^10^^-^^12^ and no reports in Asia. The follow-up study of a general population in Denmark^10^ showed the incidence rate per 100 person-years of 0.91 in both sexes, but did not report sex-specific rates. In the study of a general Norwegian population,^11^ the incidence rate with 40 incidence cases of COPD was 0.96 in males and 0.40 in females. In the study of Sweden,^12^ an average annual incidence per 100 persons of GOLD-defined COPD 0 stage cohort (a symptomatic population) with 127 incidence cases of COPD was 0.82. Thus, the incidence rate of COPD in our study was relatively similar to those reported in the previous three studies.

Current smokers were 43.3% of males and 5.5% of females in our subjects. Among males, the proportion of current smokers was higher in our study than 31.0% in the Norway study and 27.8% in the Sweden study. Among females, the proportion was lower in our study than 24.0% in the Norway study and 24.2% in the Sweden study. The proportion was 69% in both males and females in the Denmark study. There were some differences in characteristics of population such as smoking proportion between our study and the above three studies. The incidence rate of COPD among non-smokers, although not reported in other studies, would be useful for comparing with populations with different smoking proportions.

We found a statistically significant association between COPD incidence and age in males as well as in females. Among non-smokers, incidence rate of COPD was low for the age groups of 25-49 years, and gradually increased with age for the age groups of 50-74 years. This association confirms that pulmonary function decreases with age.^16^^-^^18^ The previous studies provided a significant association for all males and females and non-smokers and smokers but did not examine those groups separately. This association was confirmed by our study and described in detail.

Our study showed that COPD incidence was significantly associated with smoking status in males. This finding agrees with the relation between pulmonary function and smoking,^16^^,^^19^^-^^21^ and is consistent with those reported in the previous studies. The IRR (95% CI) of COPD for current smokers versus non-smokers was 2.0 (1.6-2.6) in the Denmark study, 9.6 (3.6-25.2) in the Norway study, and 4.6 (2.7-7.8) in the Sweden study. In our study, the IRR (95% CI) for current smokers with BI <400, BI 400-799, and BI 800+ was 1.2 (0.8-1.9), 2.7 (1.9-3.8), and 4.6 (3.3-6.5) among males, respectively. These findings indicated that smoking was a strong risk factor for COPD incidence, and that there was a clear dose-response relationship between smoking and COPD incidence. We failed, however, to detect any significant association between COPD incidence and smoking among females mainly because of the only 5.5% female smoker proportion.

There are several limitations and problems in our study. The GOLD criterion with diagnosis of COPD for FEV_1_/FVC < 70% is recognized as a standard, and was used in our study as well as in the previous studies. It might be important for diagnosing COPD to examine measures other than FEV_1_/FVC such as some definitions for airway obstruction and symptoms or histories related to COPD. An incidence of COPD was determined as when a person had been diagnosed as not COPD from measurements of FEV_1_ and FVC at baseline but was later diagnosed as COPD from those measurements during the follow-up period. In general, this method for determining an incidence of COPD is used in follow-up studies with the GOLD criterion. Spirometry is the most reproducible, standardized, and objective way of measuring airflow limitation. Although spirometry was performed by trained technicians in our study, its measurements would inevitably include variations. Our subjects for follow-up of COPD incidence might include some potential COPD cases at baseline. We might also fail to detect some potential incidence cases during the follow-up period. Most subjects received spirometry about once a year during their follow-up period. According to this follow-up method, the date when incidence cases developed COPD was not precisely identified. The vagueness in incidence date for 466 COPD cases, however, would not affect person-years in the follow-ups for 17,106 whole subjects and incidence rates of COPD. A large number of participants with only one health check-up and no information of follow-up were excluded. Although some subjects had a short follow-up period, the incidence rate did not change so much in terms of the follow-up period. Our subjects were participants in a health check-up of a community medical center at an area in Japan rather than being randomly selected from a community population, and they did not include many female smokers because of the relatively low smoking proportion among Japanese females.
